# Soft stop on syringing and probing may have a high false-positive rate in diagnosing pre-sac obstruction

**DOI:** 10.1007/s10792-022-02510-3

**Published:** 2022-09-14

**Authors:** Eiman Usmani, Yinon Shapira, Carmelo Macri, Garry Davis, Dinesh Selva

**Affiliations:** 1 Discipline of Ophthalmology and Visual Science, University of Adelaide, Adelaide, SA Australia; 2 Department of Ophthalmology, Royal Adelaide Hospital and South Australian Institute of Ophthalmology, Adelaide, SA Australia

**Keywords:** Epiphora, Lacrimal probing, Lacrimal syringing, Soft stop, Canalicular block, Dacryocystography

## Abstract

**Purpose:**

To determine the diagnostic value of 'soft stops' encountered during lacrimal syringing and probing.

**Methods:**

Single-center retrospective review. Adult patients with epiphora attending a tertiary lacrimal clinic from May 2010 to April 2021 were reviewed. Cases with evidence of soft stop encountered during lacrimal syringing/probing were included, and patients with possible canaliculitis or a history of lacrimal surgery were excluded. Findings of syringing/probing consistent with pre-sac obstruction were correlated with dacryocystography (DCG) and surgical findings.

**Results:**

53 (10.2%) canalicular systems had soft stops on syringing/probing and were included in the analysis. The mean age of the patients was 63.8 ± 15.6 (range 28–87) years, and 27 (65.9%) were females. Intraoperative examination findings were available for 27 of 30 cases that underwent lacrimal surgery and DCG was available for 40 systems. Pre-sac obstruction found on syringing/probing was confirmed in 40% and 37% of cases on DCG and surgery, respectively. The correlation between syringing/probing and DCG was stronger for canalicular than for common canalicular location (*p* = 0.016). Canalicular stenosis on syringing/probing manifested as pre-sac abnormality on DCG in 5/7 (71.4%) compared to 0/6 common canalicular stenosis cases (*p* = 0.021). Based on the surgical findings, the false-positive rate of a soft stop on syringing/probing was highest for common canalicular ‘stenosis’ (100%) and lowest for canalicular ‘block’ (45.5%; *p* = 0.093). Findings of pre-sac obstructions on DCG were confirmed in 85.7% of the cases intraoperatively (*p* = 0.035 compared to syringing/probing alone).

**Conclusions:**

Soft stops on probing showed poor correlation with DCG and surgical findings, particularly in common canalicular location.

**Supplementary Information:**

The online version contains supplementary material available at 10.1007/s10792-022-02510-3.

## Introduction

Canalicular and common canalicular pathologies (obstruction/stenosis) are found in 4.2–11% of cases of epiphora, depending on the population [1–3]. Accurate diagnosis is not always straightforward, and no gold standard method exists [4–7]. The diagnosis is generally made by demonstrating a ‘soft stop’ with either a Bowman’s probe or lacrimal cannula in clinic [8, 9]. Lacrimal imaging such as dacryocystography (DCG) is used less frequently [10].

Previous studies showed that syringing/probing is associated with low sensitivities in detecting pre-sac pathology (i.e., high false-negative rate) [4, 8, 11–13]. Nonetheless, it is unclear how accurately a soft stop on syringing/probing can predict a pre-sac etiology. Namely, the false-positive rate of syringing/probing for diagnosing a pre-sac etiology remains to be investigated.

Hence, this study aimed to correlate findings of lacrimal syringing/probing consistent with pre-sac pathology to DCG and surgical findings.

## Methods

A retrospective review was conducted of consecutive adult patients attending the Royal Adelaide Hospital lacrimal clinic with a recorded soft stop during syringing/probing between May 2010 and April 2021. Cases identified in the clinic as possible canaliculitis or patients with a history of lacrimal surgery were excluded. The study received Institutional Review Board (IRB) approval and adhered to the tenets of the Declaration of Helsinki.

### Procedures

#### Syringing/probing

Oculoplastic surgeons performed syringing, all trained under the last author (DS), who supervised and validated the technique. Lateral tension was applied to the eyelid, while a lacrimal cannula on a 2 ml syringe was inserted 1–2 mm vertically through the punctum. The lacrimal cannula was advanced slowly through the canaliculus until it reached a soft or hard stop. A ‘soft stop’ is defined as resistance to progression of the cannula by soft tissue. The cannula was then retracted and held in place, while syringing was performed with minimal pressure. The site of the soft stop from the punctum in mm, location of reflux (same, opposite, or both puncta), and subjective estimation of the degree of reflux (%) were documented.

Based on the recorded soft stop distance, ≤ 10 mm from punctum and reflux from the same punctum was designated as canalicular stenosis or block and > 10 mm, and reflux from the opposite punctum was designated as common canalicular stenosis or block.

A complete 'block' was defined as 100% reflux on syringing, whereas partial patency to the nose was defined as ‘stenosis. ’

#### DCG Technique

DCGs were performed with patients in the supine position. A drop of topical anesthetic (1% tetracaine hydrochloride) was instilled into the inferior conjunctival fornix of both eyes. The punctum was cannulated with a 27-gauge lacrimal cannula. Baseline X-ray images were taken, followed by real-time imaging during injection of contrast (Iopromide, Ultravist® 370; Bayer HealthCare Pharmaceuticals, Germany) through the cannula. This allowed digital subtraction of the pre-contrast image from post-contrast images.

Canalicular ‘stenosis’ was defined as reflux of contrast medium through the intubated canaliculus and drainage to the nasal cavity. Common canalicular ‘stenosis’ was defined as reflux of contrast medium through the non-intubated canaliculus and drainage to the nasal cavity. Furthermore, pre-sac stenosis appeared as a narrowing of the canaliculus or common canaliculus or even as a defect [14]. Complete ‘block’ was defined as no patency of contrast to the sac.

#### Surgical findings

Surgical findings were obtained from the patients' operative records. All lacrimal procedures were carried out or supervised by experienced oculoplastic surgeons (DS, GD). External or endoscopic dacryocystorhinostomy (DCR) (with or without tubes) or lacrimal (silicone) intubations were performed under general or local anesthesia with sedation. These procedures were occasionally combined with dacryoendoscopy and/or canalicular trephination, as detailed in Supplementary Table 1.

Intraoperative canalicular probing was performed in all procedures to confirm (or rule-out) pre-sac pathology. In cases that DCR was performed (external or endoscopic), direct visualization of the common internal canaliculus was recorded. Dacryoendoscopy was combined in 7/10 of the lacrimal intubations and 1/14 of the endoscopic DCRs (Supplementary Table 1).

### Statistical analysis

Data were analyzed by the StatSoft Statistica software, version 10 (StatSoft, OK, USA). Correlations between a soft stop on lacrimal syringing/probing and DCG findings, between a soft stop on lacrimal syringing/probing and surgical findings, and between DCG and surgical findings were calculated and are presented as proportions. Differences in proportions (i.e., correlations) were compared by the chi-square or Fisher's exact tests, as appropriate. A two-sided p value of less than 0.05 was considered significant.

The surgical findings (i.e., confirmation or rule-out of pre-sac pathology) were considered the gold standard for establishing the false-positive rate and the positive predictive value (PPV) of the diagnostic tests (i.e., syringing/probing or DCG). The false-positive rate denotes the proportion of soft stop cases on syringing/probing or the proportion of cases with a pre-sac abnormality on DCG, which had a normal canalicular system intraoperatively, respectively. The PPV denotes the proportion of soft stop cases on syringing/probing or the proportion of cases with a pre-sac abnormality on DCG, which was confirmed to have a pre-sac etiology intraoperatively, respectively.

## Results

From May 2010 to April 2021, syringing/probing was available for 519 symptomatic lacrimal systems. Out of these, 62 (11.9%) soft stops in 49 patients (bilateral in thirteen) were documented. In nine of these cases, the soft stop was noted to have been overcome on subsequent syringing/probing performed in the clinic, and they were thus excluded from the analysis [i.e., 53 (10.2%) soft stops in 41 patients were included].

**Fig. 1:**
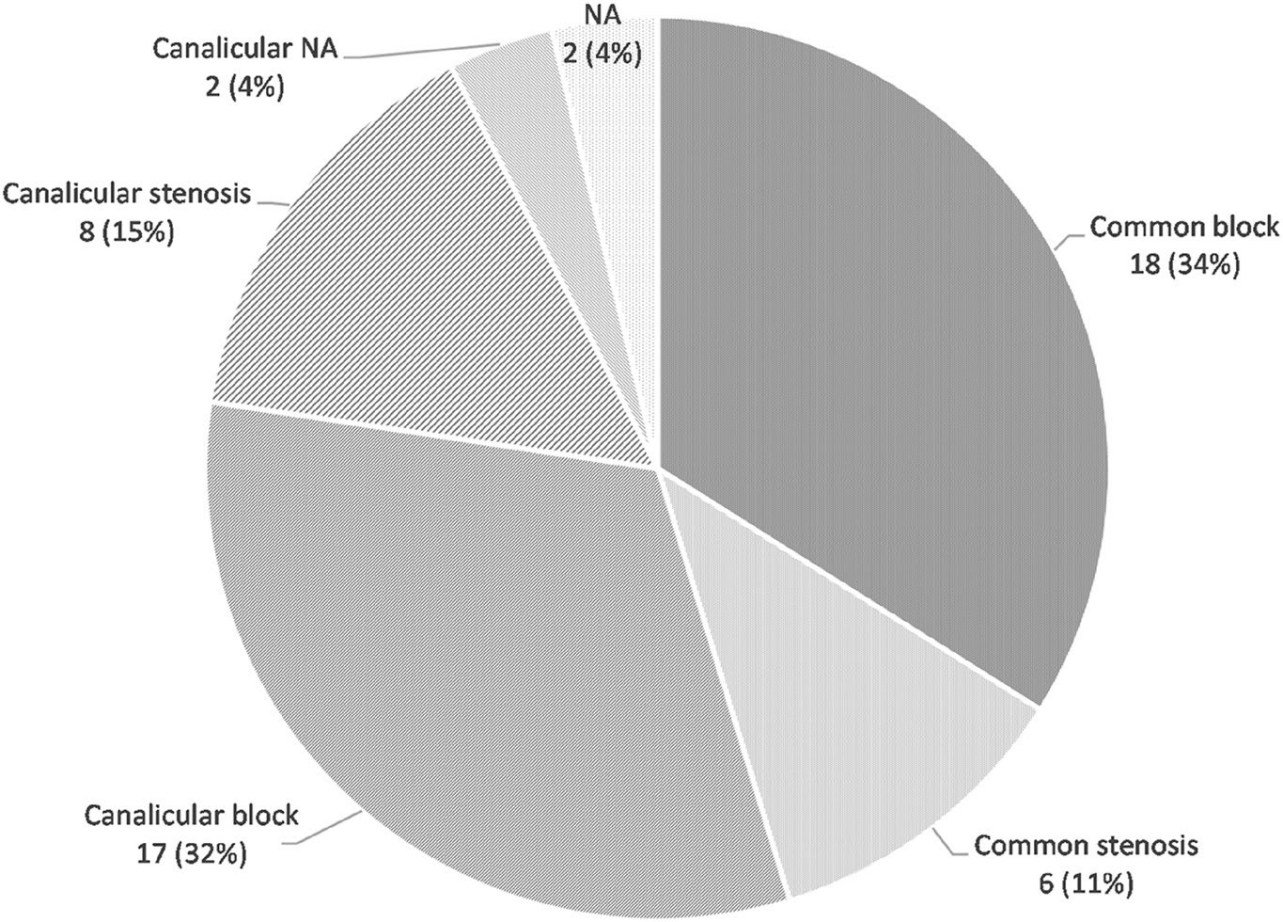
Location and degree (stenosis or block) of pre-sac obstruction based on lacrimal syringing/probing in the clinic. NA obstruction location and/or degree not available

The mean age of the patients with a soft stop was 63.8 ± 15.6 (range 28–87) years, and 27 (65.9%) were females. Based on the syringing/probing, the location of obstruction was the canaliculus in 27 (50.9%), common canaliculus in 24 (45.3%), and unavailable for 2 (3.8%; Fig. 1).

### Correlation between syringing/probing and DCG

DCG was available for 40 (75.5%) lacrimal systems with a soft stop on probing. The correlation between the syringing/probing and the DCG is shown in Table 1. Pre-sac abnormality (i.e., stenosis or obstruction) was confirmed on DCG in 16 (40%) cases. In 10 (25%) cases, post-sac abnormality was found, and in 14 (35%), the DCG was normal (Table 1).

**Table 1:** Correlation between a soft stop on lacrimal syringing/probing and DCG findings

Syringing/probing	DCG abnormality			No abnormality
	Canalicular	Common canaliculus	Post-sac	
Canalicular block (*n* = 11)	5	1	2	3
Canalicular stenosis (*n* = 7)	3	2	0	2
*Total canalicular*^a^* (n* = *20)*	*9 (45%)*	*3 (15%)*	*2 (10%)*	*6 (30%)*
Common canaliculus block (*n* = 12)	1	2	6	3
Common canaliculus stenosis (*n* = 6)	0	0	1	5
*Total common canalicular (n* = *18)*	*1 (5.6%)*	*2 (11.1%)*	*7 (38.9%)*	*8 (44.4%)*
*Total with soft-stop*^b^* (n* = *40)*	*10*	*6*	*10*	*14*

*DCG* Dacryocystography

^a^Including canalicular soft stop cases with unavailable degree (stenosis or block) of obstruction

^b^Including soft stop cases with unavailable location or degree of obstruction

Highlights the total number of cases (stenosis + block)

The correlation between syringing/probing and DCG was stronger for canalicular than for common canalicular location (*p* = 0.016, Table 1). This was most notable for stenosis cases, as canalicular stenosis on syringing/probing manifested as pre-sac abnormality on DCG in 5/7 (71.4%) compared to 0/6 common canalicular stenosis cases (*p* = 0.021, Table 1).

### Correlation with the surgical findings

Supplementary Table 1 outlines the management course of soft stop cases (including surgical procedures). The intraoperative canalicular examination was available for 27 of the 30 soft stop cases that underwent a lacrimal procedure.

A pre-sac etiology was confirmed intraoperatively in 10/27 cases, representing a 37% general positive predictive value (PPV) of a soft stop on preoperative syringing/probing. Depending on the soft stop location on preoperative probing, 7/14 (50%) of canalicular and 3/13 (23.8%) of common canalicular soft stops were confirmed intraoperatively as a pre-sac etiology (*p* = 0.24). More specifically, 6/11 (54.5%) of canalicular blocks, 1/3 (33%) of canalicular stenoses, 3/8 (37.5%) of common canalicular blocks, and 0/5 of common canalicular stenoses on syringing/probing were confirmed intraoperatively.

Based on the surgical findings, the false-positive rate of a soft stop on syringing and probing was highest for common canalicular stenosis (100%) and lowest for canalicular block (45.5%); however, the difference did not reach statistical significance (*p* = 0.093) due to the low number of cases in the subgroups.

Preoperative DCG was available for 21 of the 27 soft stop cases with an intraoperative examination. A pre-sac etiology was confirmed intraoperatively in 6/7 of the DCG cases showing a pre-sac abnormality (*n* = 2 canalicular block, *n* = 3 canalicular stenosis, *n* = 1 common canalicular block on DCG). Thus, representing an 85.7% general PPV of pre-sac abnormality on DCG (*p* = 0.035 compared to syringing/probing alone). On the imaging, the single false-positive DCG (14.3%) appeared as common canalicular stenosis (combined with a post-sac block) and was noted to have a normal canalicular system intraoperatively. There was a 1/7 (14.3%) false-negative rate for the DCG, namely a post-sac stenosis on DCG that was found intraoperatively to have canalicular stenosis.

## Discussion

In the current study, the proportion of epiphora patients with pre-sac pathology diagnosed based on clinical examination was similar to previous reports (10.2%) [1–3]. However, pre-sac obstructions encountered during syringing and probing were confirmed only in 37% of cases intraoperatively. Furthermore, syringing/probing demonstrated a low concordance rate (40%) with DCG, and the addition of DCG provided a significantly higher positive predictive value (85.7%). Therefore, our study suggests that canalicular and common canalicular pathology are overestimated during routine syringing and probing in the clinic, even in experienced hands.

In our cohort, 45% of cases were diagnosed with common canalicular obstruction during syringing/probing. These cases showed the lowest correlation with both DCG and surgical findings. Furthermore, the agreement between syringing and DCG/surgery was greater for canalicular than common canalicular obstructions. This was most striking for the common canalicular stenosis group (100% false-positive rate using DCG and intraoperative results).

There are plausible explanations for the high false-positive common canalicular soft stops on syringing/probing. Firstly, common canalicular/lacrimal sac mucosal folds (CLS-MFs) have been shown to impact the internal canalicular orifice on endoscopy and falsely suggest an obstruction during probing [15, 16]. These mucosal folds occur in roughly 60% of the population [11, 17–20]. Next, changes in angulation within the course of the canaliculi can lead to kinking and the impression of a false soft stop [19, 21, 22]. It is plausible that kinking is more likely to occur distally along the canaliculus.

Previous studies emphasized the inaccuracy of syringing and probing for diagnosing a pre-sac etiology [8]. In studies by Boboridis et al. [11] and Irfan et al. [13], preoperative syringing and probing failed to identify 43% and 53% of pre-saccal blocks identified during surgery, respectively. Furthermore, distinguishing between canalicular stenosis and obstruction is unreliable using standard syringing/probing technique [4]. While these studies highlight that syringing/probing is associated with high rates of missing pre-sac obstructions (low sensitivity), our study adds that syringing and probing also have a high false diagnosis rate as a stand-alone test, particularly for common canalicular obstruction.

DCG was previously shown to be sensitive in detecting canalicular pathology [13, 23–25], consistent with the current study. DCG showed a high correlation with both syringing/probing and surgical findings in diagnosing canalicular stenosis. On the other hand, there was a single case of false-positive common canalicular stenosis on DCG. CLS-MFs may appear as an area of common canalicular stenosis on DCG [26], and distal kinking of the canaliculus may manifest as a pseudo-obstruction [27]. Taken together, these results suggest that there may be value in confirming equivocal syringing/probing findings on DCG.

Limitations to this study firstly include its retrospective nature. Secondly, some trends did not reach significance due to a small cohort. This was because suspected canalicular and common canalicular pathologies formed a very small proportion in our studied population over ten years. This is in contrast to some other populations where a pre-sac etiology was a more common finding [28]. Furthermore, this study focused only on cases with presumed pre-sac stenosis/obstructions. Thus, the findings do not reflect the test's sensitivity but only its predictive value. Finally, syringing/probing is operator-dependent, and while the same technique and standards were assured over the study period, inter-tester variations may have been introduced over the years.

In conclusion, our results demonstrated a low predictive value of a soft stop in diagnosing a pre-sac etiology for epiphora. Clinicians should be aware of this when consenting patients for surgical intervention and be prepared to alter their surgical approach if preoperative findings are not confirmed intraoperatively. Clinicians may also consider performing dacryocystography to improve diagnostic accuracy for common canalicular pathology.

## Supplementary Information

Below is the link to the electronic supplementary material.

Supplementary file1 (DOCX 24 kb)
