# Supplementary material for: Soft stop on syringing and probing may have a high false-positive rate in diagnosing pre-sac obstruction
Source: Int Ophthalmol. 2022 Sep 14;43(4):1127–33. doi: 10.1007/s10792-022-02510-3 (PMC10113285; doi:10.1007/s10792-022-02510-3)
Supplement: Supplementary file 1 — Supplementary file1 (DOCX 24 kb) [file 10792_2022_2510_MOESM1_ESM.docx]

Supplementary Table 1: Management course of 53 cases with a soft stop on lacrimal syringing and probing

| Lacrimal procedure (n=30) |  | *With dacryoendoscopy* | *With canalicular trephination* | *With tubes* |
| --- | --- | --- | --- | --- |
|  | External DCR (n=5) | 0 | 1 | 4 |
|  | Endoscopic DCR (n=14) | 1 | 3 | 7 |
|  | Lacrimal intubation (n=10)^1^ | 7 | 4 | 8 |
|  | Canalicular cut down (n=1) | 0 | 0 | 0 |
| No lacrimal procedure (n=23) | Eyelid procedure (n=3) | | | |
|  | Declined procedure (n=5) | | | |
|  | Conservative/lost to FU (n=15) | | | |

^*^ Dacryocystorhinostomy (DCR)

† n=2 dacryoendoscopy without placement of tubes (n=1 inability to pass tube, n=1 decision not to intubate due to normal exam
